# The status and challenges of HPV vaccine programme in China: an exploration of the related policy obstacles

**DOI:** 10.1136/bmjgh-2023-012554

**Published:** 2023-08-16

**Authors:** Huizi Wang, Yujin Jiang, Qing Wang, YuetMan Lai, Aisha Holloway

**Affiliations:** 1 Department of Nursing, The First Affiliated Hospital of Shandong First Medical University & Shandong Provincial Qianfoshan Hospital, Jinan, Shandong, China; 2 School of Health in Social Science, The University of Edinburgh, Edinburgh, UK; 3 Operating Room, Shandong Province Maternal and Child Health Care Hospital, Jinan, Shandong, China; 4 Department of Outpatient, Shandong Cancer Hospital and Institute Shandong First Medical University and Shandong Academy of Medical Sciences, Jinan, Shandong, China

**Keywords:** health policy, health insurance, health education and promotion, vaccines, child health

**Summary box**

- China has a high burden of human papillomaviruses (HPV)-related cancers, with the second highest incidence and mortality rate of cervical cancer worldwide.
- Financial constraints, regional restrictions and gender-specific vaccination policies have led to disparities in HPV vaccination rates across China.
- Public perceptions of the HPV vaccine vary widely, with factors such as lack of confidence, accessibility and awareness influencing vaccine uptake.
- Recommendations include increasing government funding for the HPV vaccine, expanding vaccine coverage, improving vaccine delivery yield, regulating injection pathways and enhancing scientific education on the HPV vaccine to raise awareness and increase vaccine uptake.

## Introduction

Human papillomaviruses (HPV) are DNA viruses with a double strand of DNA that belong to the family Papovaviridae.^1^ Currently, nearly 200 HPV types have been identified and are categorised into two groups based on their carcinogenicity, high risk and low risk.^2^ Among them, high-risk HPV (HR-HPV) types 16, 18, 31, 33, 35, 39, 45, 51, 52, 56, 58, 59 and 66 are the most concerning, as they are associated with cancer development.^3^ It is estimated that HR-HPVs cause about 90% of cervical cancer cases and an increasing number of head and neck cancers.^4^

Globally, cervical cancer is the most common cancer associated with HR-HPVs and ranks fourth among women.^5^ Almost all cervical cancer cases are caused by HR-HPVs infections.^6^ Given that China is the world’s largest and most populous low-income and middle-income country (LMIC), there is a substantial burden of cervix cancer.^7^ According to the latest global cancer burden study, China has the second highest incidence and mortality rate of cervical cancer worldwide, with around 110 000 new cases and 59 000 deaths in China.^8^ There is no doubt that cervix cancer remains a major public health issue in China. Furthermore, over the past few decades, a growing number of studies show that certain head and neck cancers (HNCs), mainly oropharyngeal cancers, are associated with HR-HPVs, especially type 16.^9 10^ Sabatini and Chiocca^9^ indicate that there has been a 37% increase in HNCs incidence globally over the past decade. The Global Cancer Statistics 2020 report highlights that HNCs of the lip, oral cavity, larynx and pharynx collectively rank among the top 10 most common adult cancers globally, comprising around 880 000 cases and 440 000 fatalities.^11^ Moreover, the overall incidence of HNC is expected to continue to rise, with a projected 30% annual increase by 2030, particularly in developing countries.^12^ According to data from the National Cancer Center Registry in China, HNC caused above 230 000 new cases and 78 000 deaths.^13^ In comparison to other cancers, HNC has a very low prognosis and a poor 5-year survival rate.^14^ HPV is a major cause of cervical cancers as well as a significant proportion of oropharyngeal, vaginal, vulvar and penile cancers.^15^ But as a group of cancer types with a known aetiology, countries would be able to eradicate HPV-related cancers if both men and women received universal vaccination.^15 16^ HPV vaccines are effective against the majority of high-risk subtypes of HPV,^17^ with three main types of vaccines currently produced globally: bivalent (covered HPV 16, 18), quadrivalent (covered HPV 6, 11, 16, 18) and 9-valent HPV (covered HPV 6, 11, 16, 18, 31, 33, 45, 52, 58) vaccines.^17^ In several countries, specifically LMICs, HPV vaccination is still relatively new.^18^ According to 2020 statistics, the proportion of women aged 9–45 in China who had received a complete HPV vaccination was only 3%.^19^ To expand HPV vaccine coverage and responded to the ‘Accelerated Eradication of Cervical Cancer’ initiative proposed by the WHO, China has put forward national policies such as ‘Healthy China Action (2019–2030)’, which includes provisions for intensify public education and publicity on the HPV vaccine. It also aims to promote vaccination in the target age group,^20^ with HPV vaccination currently being launched in 15 pilot cities for girls aged 13–15 years (see table 1).^21^

**Table 1:** List of the first pilot cities for HPV vaccine in China （translated from the Office of the National Patriotic Health Campaign Committee)

Number	Province	City (District)
1	Beijing	Shijingshan district
2	Tianjin	Xiqing district
3	Neimenggu	Eerduosi
4	Liaoning	Shenyang
5	Shanghai	Minhang district
6	Jiangsu	Wuxi
7	Zhejiang	Ningbo
8	Anhui	Maanshan
9	Fujian	Xiamen
10	Shandong	Jinan
11	Henan	Zhengzhou
12	Guangdong	Shenzhen
13	Chongqing	Shapingba distinct
14	Sichuan	Chengdu
15	Shanxi	Xian

The policy adopted by China aligns with the WHO’s guidelines for standard HPV vaccination among those aged 9–14.^22^ However, China’s vaccination programme only covers limited age groups, leaving those not included with no specific solutions. Additionally, notable differences in awareness and understanding of the HPV vaccine exist in China, driven by the multiplicity and disparity across regions and socioeconomic classes.^23^ To complete the HPV immunisation programme successfully, China must overcome the challenge of ensuring individuals of all ages receive the optimal HPV vaccine at the right time and have a thorough understanding of the vaccine.

## Concerns

### Narrow scope of HPV vaccination programme in China

China’s HPV vaccination programme was developed and launched later and more slowly than other developed countries. In China, the existing policy on HPV vaccinations only grants free vaccinations to girls aged 13–15 in select regions,^24^ with limitations based on age, gender and geographical location. The age restrictions on HPV vaccination can result in a missed optimal vaccination period for some individuals, as HPV infection peaks among Chinese women occurs between the ages of 20 and 24,^25^ a time when many are attending college or university. Therefore, university students are at the high-risk of HPV infection.^26^ However, HPV vaccination is not included in China’s national vaccination programme and individuals want to get vaccinated must pay out of pocket (OOP).^27^ Currently there are five HPV vaccines approved by the China Food and Drug Administration, including the bivalent domestic vaccine Cervarix, the bivalent imported vaccine Cecolin and Walrinvax, the quadrivalent HPV vaccine Gardasil 4, the nine-valent HPV vaccine Gardasil 9. These vaccines cost between US$140 and US$580.^27^ And college students, who often have no fixed income, may not be able to afford vaccination due to a lack of funding. Similarly, region-based restrictions may result in people in remote and poor areas not having additional funds for this preventative vaccination, which has contributed to health inequalities in less underdeveloped regions. The restriction of HPV vaccination based on gender may mask the commonality of HPV in sexually active men.^28^ In developed countries where male vaccination has been introduced, cost-effectiveness models have demonstrated the superiority of including male vaccination.^29^

Currently, public health funding in China is relatively low.^30^ Despite studies showing that Chinese government spending on healthcare has quadrupled in the last decade, Chinese OOP spending on healthcare has fallen dramatically, especially for catastrophic illnesses.^31 32^ The lack of a clear and consistent government subsidy scheme for preventive immunisation vaccines, such as the HPV vaccine, remains a challenge in China. However, cervical cancer has resulted in a substantial economic burden in China, with the average cost of cervical cancer-related expenses ranging from US$7400 to US$22 000 per person during the year following their final discharge.^33^ In addition, the hospital costs for patients receiving head and neck cancer surgery range from US$520 to US$3100.^34^ However, in 2022, China’s per capita disposable income is US$5200.^35^ Therefore, suffering from HR-HPVs-related cancer can be a significant financial burden for individuals and families. These health and economic burdens can be largely preventable with already available vaccines. The latest systematic review and meta-analysis including data from 60 million individuals and up to 8 years of HPV vaccination shows that HPV vaccination programme have a dramatic impact on girls and women’s resistance to HR-HPVs, with an 83% increase for girls aged 13–19 years and a 66% increase for women aged 20–24 years.^36^ Furthermore, the effectiveness and extensive coverage of a vaccination programme provide protection not only to the vaccinated but also aid in preventing cross-protection and herd effects in boys and older women.^37^ As such, boosting financial support for HPV vaccines to achieve complete immunisation, defined as the individual receives the recommended dose of HPV vaccine under the national immunisation programme, is a crucial means of effectively decreasing the incidence of HPV-related cancers and the socio-economic burden they impose.^38^

### Disparities in public perceptions of the HPV vaccine

As a class II vaccine with a commercial character, the HPV vaccine is available in China; however, the decision to receive the vaccine ultimately rests on the individual’s volition.^39^ According to recent studies, the acceptance rate of the HPV vaccine among mainland Chinese individuals varies widely, ranging from 35% to 90%.^40^ This broad range suggests that there may be significant differences in attitudes and beliefs about the vaccine, and underscores the importance of understanding the factors that influence vaccine uptake in this population.

Given that the recommended age for HPV vaccination falls within the category of minors, obtaining parental consent in addition to the individual’s permission is necessary.^41^ In traditional Chinese parent–child families, where parents make most of the decisions, parental attitudes and understanding are crucial.^42^ Nevertheless, various studies have revealed that most Chinese parents exhibit vaccine hesitancy, primarily due to a lack of confidence and accessibility to vaccines.^43 44^The lack of confidence in vaccines is exemplified by questions regarding safety and efficacy. Due to limited availability of the HPV vaccine in China, numerous parents hold the view that there is insufficient evidence to verify its effectiveness and safety without side effects.^45^ Furthermore, previous incidents of vaccine safety issues in China, such as the Changchun Changsheng Biotechnology Company’s rabies vaccine falsification scandal in 2018, have significantly diminished public trust, resulting in lower vaccination rates and an increase in vaccine hesitancy.^46^ The lack of accessibility is apparent from the fact that, apart from the 15 pilot cities where HPV vaccination is free for school-age adolescents, parents are required to pay for their children to receive the HPV vaccine. Although parental acceptance of the HPV vaccine is relatively high, when it comes to self-payment, only 56% of parents accept the price of <US$140.^43^ Therefore, in addressing parental vaccine hesitancy, Chinese public health authorities must prioritise increasing public trust in the HPV vaccine and finding ways to make it more affordable.

As for Chinese adult women, awareness of the HPV vaccine is mostly driven by socio-demographic factors. More specifically, individuals living economically advanced regions are inclined to pursue high-valent imported vaccines as they prioritise quality and effectiveness.^47^ For instance, the 9vHPV vaccine has been shown to be 98% effective against HPV-6/11/16/18/31/33/45/52/58 related cervical, vulvar and vaginal disease, compared with 2vHPV which was 93% effective only against HPV-16/18 related infections,^48^ and approximately 90% effectiveness of 4vHPV in reducing the incidence of HPV-6/11/16/18 infections and condyloma acuminata.^49 50^ According to statistics from one developed city in eastern China, demand for the 9-valent HPV vaccine exceeds supply, for which many e-commerce platforms have launched booking packages, charging even more than US$140 for the booking service.^51^ In contrast, women in less economically developed regions, such as western China, are generally unaware of the importance of HPV vaccination, which is mainly attributed to the low level of awareness of HPV and HPV vaccine.^52^ In addition, a systematic review of 73 literatures examining HPV vaccine acceptability in China yielded results that were consistent with the authors’ hypothesis and indicated that awareness of the HPV vaccine varied by geographical region.^40^ In general, improving the supervision of China’s drug regulatory authorities, regulating the sales chain of vaccine agents and distributors and promoting basic knowledge of HPV and HPV vaccine in less developed areas are the main issues that need to be addressed at this stage.

Even though different groups hold different views about the HPV vaccine, Loke *et al*,^53^ indicate that healthcare professionals (HCPs) are essential to promoting uptake, especially among adolescents. Generally, HCPs with expertise in HPV vaccination are more likely to provide strong HPV vaccine advice.^54 55^ Similar findings have been observed for other vaccines, such as hepatitis A and hepatitis B, in which HCPs with extensive knowledge tend to recommend them, leading to higher vaccination rates among children.^56^ Therefore, it is necessary to consider strategies to improve medical knowledge about the HPV vaccine among all healthcare workers and to improve access to healthcare services.

## Conclusion

The available evidence highlights the importance of increasing financial investment in immunisation, particularly in HPV vaccines, and enhancing the regulatory oversight by China’s drug authorities to monitor marketing approaches. In addition, it is essential to develop a comprehensive HPV vaccine promotion plan, especially launching educational programmes aimed at raising awareness and knowledge of HPV infection and vaccination in the community and rebuilding public confidence in domestically produced vaccines.

## Recommendations

### Step up government funding for the HPV vaccine

To further improve the population’s public health, it is recommended that the government increase its expenditure. The implementation of national vaccine immunisation, which is one of the most cost-effective health interventions, should be prioritised. In view of China’s status as an LMIC, achieving universal HPV vaccination among school-aged men and women over time may be an uphill battle. To surmount this challenge, the authors advise that China take a cue from developed Western nations and prioritise the inclusion of women of the relevant age group in the free HPV programme during its initial stages of implementation. Furthermore, a reduced vaccination dose should be considered. In 2022, the Strategic Advisory Group of Experts on Immunization recommended girls and women aged 9–14 and 15–20 receive one or two doses and noted that a single dose is comparable to a two-dose schedule.^18^ In comparison to a two-dose or three-dose HPV vaccination schedule, a one-dose vaccination would address various hurdles, such as reduced project costs and ease of management.^18^

Alternatively, choosing the domestic HPV vaccine as the first choice is another feasible alternative. As it is half the price of the imported vaccine, which makes it more accessible to a larger population, even with limited funding. For certain provinces and cities that do not yet have sufficient funds to provide free vaccinations, innovative pricing and financing mechanisms can be used to alleviate the issue of high vaccine prices. For example, in Suzhou, China, parents can now pay for vaccines through their health insurance accounts, which addresses some parents’ vaccine hesitations.^57^

### Increasing vaccine delivery yield and regulating injection pathways

With the widening of the age of 9vHPV vaccination to 9–45 years in China, more and more people are opting for this more protective vaccine. It has become evident that demand for the vaccine is surpassing the available supply. In this regard, Chinese biopharmaceutical companies should accelerate their research and development and clinical trials to mitigate the shortage of imported 9-valent vaccines. Chinese drug regulators can also choose to diversify their import channels by importing to regions such as Hong Kong and Macau, where demand has been relatively low. Meanwhile, regulating the high cost of HPV vaccines reservation fee and regulating the integrated service of hospital, community and school appointments and injections are recommended actions.

### Enhancing scientific education on HPV vaccine

Public health behaviour is contingent on both greater awareness of the need to prevent HPV infection and the cooperation of the government, medical institutions and educational institutions. The overall population’s rate of HPV vaccination cannot be raised unless people are educated about the significance and repercussions of HPV as well as the efficacy of the HPV vaccine.^27^

The most comprehensive information on HPV vaccine, HR-HPVs related cancer prevention is provided to HCPs at provincial, municipal and county levels, which can effectively increase health literacy of different consultation audiences (such as parents of teenagers, university students, women of school age) and increase the vaccination rate. The education department should integrate health education related to HPV infection into the curriculum of middle and high schools and colleges. And health promotion activities can be conducted regularly in collaboration with HCPs. Previous studies in the USA have shown the beneficial effects of educational interventions delivered by healthcare providers on vaccine education and adherence.^58 59^

On the other hand, exploring the online platform of sustainability services can facilitate access to professional answers whenever people have questions. In recent years, people have increasingly turned to the internet for quick answers, but without professional guidance. the information may be inaccurate. Developing a professional online inquiry platform that allows people to get answers from professionals would provide a valuable digital offering. Furthermore, HCPs can effectively disseminate information on HR-HPVs related cancers and the HPV vaccine by leveraging new media platforms such as TikTok, WeChat Public and Weibo, which are highly accessible and currently among the most effective channels for delivering health-related messages.

## Data Availability

Data are available in a public, open access repository.
